# Influence of S100A2 in Human Diseases

**DOI:** 10.3390/diagnostics12071756

**Published:** 2022-07-20

**Authors:** Hitomi Sugino, Yu Sawada

**Affiliations:** Department of Dermatology, University of Occupational and Environmental Health, 1-1 Iseigaoka, Yahatanishi-ku, Kitakyushu 807-8555, Japan; hsugino@med.uoeh-u.ac.jp

**Keywords:** S100A2, cancers, inflammatory diseases

## Abstract

S100 proteins are a family of low-molecular-weight proteins characterized by two calcium-binding sites with a helix-loop-helix (“EF-hand-type”) domain. The S100 family of proteins is distributed across various organs and can interact with diverse molecules. Among the proteins of the S100 family, S100 calcium-binding protein A2 (S100A2) has been identified in mammary epithelial cells, glands, lungs, kidneys, and prostate gland, exhibiting various physiological and pathological actions in human disorders, such as inflammatory diseases and malignant tumors. In this review, we introduce basic knowledge regarding S100A2 regulatory mechanisms. Although S100A2 is a tumor suppressor, we describe the various influences of S100A2 on cancer and inflammatory diseases.

## 1. Introduction

S100 proteins are a family of low-molecular-weight proteins characterized by having two calcium-binding sites with a helix-loop-helix (“EF-hand-type”) domain [1]. Because S100 family proteins are distributed across various organs and can interact with a diverse range of molecules [2,3,4,5], they play vital roles in numerous aspects of physiological and pathological events. They are currently highlighted for investigators to elucidate their role in human diseases.

Among the S100 family proteins, S100 calcium-binding protein A2 (S100A2) is encoded by a gene located on chromosome 1q21 [6]. Although S100A2 interacts with calcium, S100A2 also binds to zinc with a high affinity [7]. Zinc inactivates S100A2 function by inhibiting response to intracellular calcium signals [7]. S100A2 has been identified in mammary epithelial cells, glands, lungs, kidneys, and prostate gland [8]. Therefore, S100A2 is expected to have various physiological and pathological effects on human diseases.

Although S100A2 has a tumor suppressor function [9], the recently updated research focusing on the prognostic impact of S100A2 revealed the various influences of S100A2 in human malignancies [10,11], in addition to the inflammatory diseases [12]. In this review, we introduce basic knowledge surrounding S100A2 and its regulatory mechanisms. Furthermore, we describe in detail the influence of S100A2 on malignant tumors and inflammatory diseases.

## 2. Regulatory Action of S100A2 and Interaction with Other Molecules

Human S100A2 is an EF-hand calcium-binding S100 protein predominantly located in epithelial cells [13]. In particular, S100A2 is expressed in keratinocytes in a dimeric form in normal physical state [14]. S100A2 is predominantly expressed in the nucleus and moderately so in the cytoplasm [15]. However, this distribution pattern of S100A2 is altered under stress stimuli, such as oxidative stress, which enhances the cytoplasmic translocation of S100A2 from the nucleus [15]. This phenomenon can be observed under conditions of increased intracellular Ca^2+^ levels [15]. This cytoplasmic S100A2 is released by the leakage from the S100A2 containing cells due to cell death events. 

**Figure 1:**
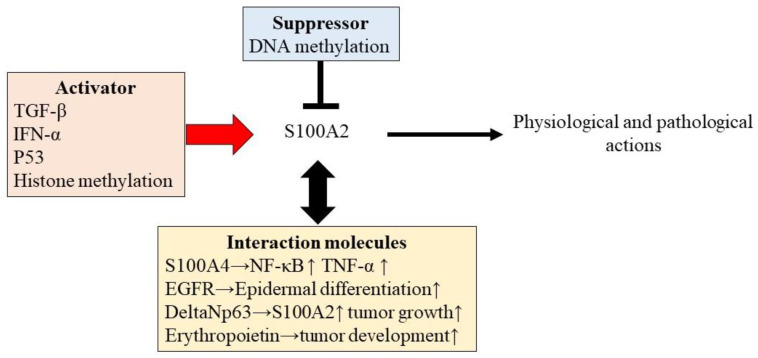
The regulation of S100A2 expression.

Various other stimuli, including transforming growth factor (TGF)-β [16] and interferon (IFN)-α [17], can also trigger modulation of S100A2 expression (Figure 1). Therefore, various inflammatory reactions appear to be associated with S100A2-mediated physiological and pathological reactions in the human body.

## 3. Interactions of S100A with Other Molecules

A recent study revealed that S100A2 interacts with various physiological regulatory factors to enhance its regulatory potential. S100A2 interacts with other S100 family proteins, such as S100A4 [18]. S100A2 interacts with S100A4 in a cross-linked manner, mediated by the copper-mediated oxidation of cysteine residues, which increases the induction of NF-κB and TNF-α production [18] (Figure 1).

S100A2 is localized to an intermediate filament cytoplasmic protein, cytokeratin K14, which is predominantly present in basal proliferative keratinocytes [19]. S100A2 is also an enhancer of epidermal differentiation in the regenerative hyperplasia pathway, which is mediated by the epidermal growth factor (EGF) receptor and/or related receptors of ErbB family-mediated pathway activation [20]. These findings suggest an important role for S100A2 in the epidermal physiology and oncogenesis of tumors derived from the epidermis.

S100A2 also plays an important role in the pathogenesis of tumors. Indeed, S100A2 is also induced by the p53-stimulator etoposide [21]. A possible binding site for p53 is located at the promoter site of *S100A2* [21]. Therefore, p53 can positively regulate S100A2 expression [21,22,23,24]. S100A2 is also a direct transcriptional target of ΔNp63alpha and is essential for keratinocyte differentiation [25]. Further, BRCA1 can interact with ΔNp63 proteins, enhancing S100A2 expression and tumor growth [26].

In contrast, erythropoietin can interact with S100A2 to enhance tumor development [27]. Since S100A2 plays a role in regulating tumor development in almost all tumors, the interaction of S100A2 with erythropoietin might be important in certain conditions, such as erythropoietin-dominant tumor development.

## 4. Epigenetic Regulation of S100A2

Epigenetic regulation has been postulated as one mechanism underlying S100A2 regulation, especially in malignancies. Epigenetic chemical modification of DNA itself or DNA-binding proteins, such as histones, changes gene expression without altering the sequence [28,29,30] (Figure 1).

Among epigenetic chemical modifications, deoxyribonucleic acid (DNA) methylation is a representative epigenetic modification [31,32,33]. CpG islands are often modified by DNA methylation, and typically influence CpG islands on the promoter sites of target genes [34]. Such sites comprise a cytosine nucleotide followed by a guanine nucleotide in a linear sequence from the 5′ to the 3′ direction of the DNA region. DNA methylation essentially acts to silence targeted gene expression [35]. As CpG islands are often observed in gene promoter sites, DNA methylation is a powerful transcriptional modulation mechanism in various diseases [36]. Indeed, the hypermethylation of targeted gene promoter CpG sites is often observed in cancer cells [9,37]. In addition, targeted gene-specific DNA methylation leads to the reduced expression of S100A2 in normal cells [9], while treatment with demethylation agent 5-aza-2′-deoxycytidine can recover the expression of S100A2 [38]. These findings indicate that DNA methylation is a mechanism for regulating *S100A2* gene expression.

Histone methylation primarily targets histone H3 lysine residues, which can drive both the activation and suppression of gene transcription [28]. Histone methyltransferases enhance histone methylation, whereas histone demethylases reverse histone methylation. Lysine-specific demethylase 5C (KDM5C) is a lysine-specific histone demethylase that belongs to the Jumonji family of demethylases, and is specific for di- and tri-demethylation of lysine 4 residues on histone 3 (H3K4 me2/3) [39]. KDM5C recovers S100A2 expression through histone demethylation to impair the proliferation and migration of cancer cells, suggesting that histone methylation leads to the impairment of S100A2 expression in tumors [40].

## 5. Inflammatory Diseases

**Figure 2:**
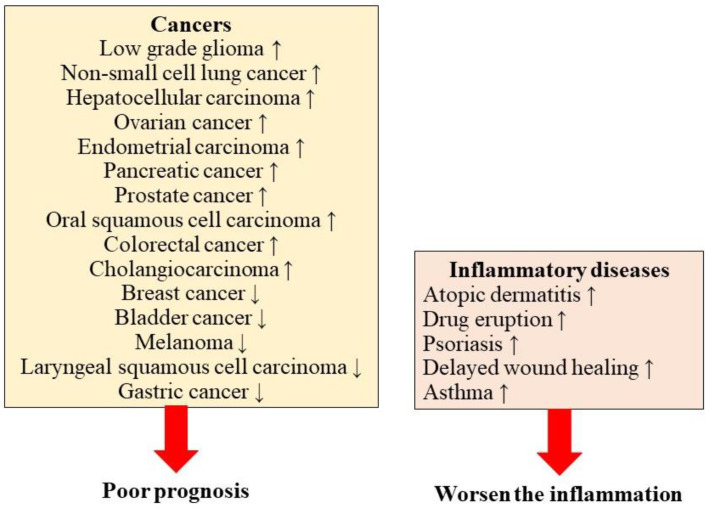
The influence of S100A2 in human diseases.

Although a limited number of studies have investigated inflammatory diseases, previous studies have speculated about the possible role of S100A2 in the development of inflammatory diseases (Figure 2).

S100A2 is highly upregulated in the epidermis under inflammatory conditions and in drug eruptions, in addition to inflammatory skin diseases such as atopic dermatitis and psoriasis [12]. An important finding is that the degree of S100A2 expression depends on the severity of inflammatory skin diseases [12]. Furthermore, epithelial-specific S100A2 transgenic mice showed the activation of the proliferation and migration of S100A2-deficient human keratinocytes [41]. In addition, S100A2-expressing mice exhibit a delayed response to cutaneous wound repair action [41]. The stress-activated p53 tumor suppressor protein plays an important role in cutaneous wound healing and is an S100A2 inducer [41]. Although it remains unclear whether S100A2 is involved in physiological function in hair follicles, S100A2 is highly expressed in the entire outer-root sheath, whereas its expression is lower in the medulla and cuticle in the bulb [42], suggesting that alopecia areata or other hair-related diseases might also be associated with S100A2-mediated pathogenesis.

The exposure of lung epithelial cells to Ni enhances S100A2 expression, suggesting that S100A2 is involved in lung inflammatory diseases [43]. Consistently, S100A2 levels are increased in the saliva of patients with uncontrolled asthma [44], suggesting that lung inflammatory diseases are also associated with S100A2-mediated pathogenesis.

## 6. Benign Tumors

Several benign tumors are known to be associated with the expression of S100A2. Eccrine poromas and syringomas express S100A2 [45], as do apocrine hidrocystomas. Syringocystadenoma papilliferum express S100A2 [45], and calcifying epitheliomas in basophilic cells exhibit positive staining for S100A2 [46]. 

Craniopharyngioma is a benign tumor that exhibits positive S100A2 expression [47]. The expression of gamma delta T cells is positively associated with S100A2. In contrast, the presence of CD8^+^ T cells is negatively correlated with S100A2 expression [47], suggesting a possible role of S100A2 in the regulation of immunological reactions.

## 7. Role of S100A2 in Malignancies

The importance of S100A2 expression has been identified in various malignancies. In this section, we describe the different roles of S100A2 in different organ-derived malignant tumors to better understand the role of S100A2 in tumor development (Figure 2).

## 8. Brain Cancer

Low-grade glioma is a progressive malignant brain tumor in which S100A2 is upregulated [48]. High S100A2 expression is significantly associated with poor prognosis in patients with low-grade glioma in vivo [48]. Furthermore, S100A2 is also epigenetically regulated by DNA methylation of CpG islands, and high S100A2 expression is related to glioblastoma tumor cell proliferation, apoptosis, invasion, and migration in vivo [49]. S100A2 expression is also related to high tumor grade and is frequently observed in high-clinical-stage gliomas. CpG methylation has been observed in gliomas, and it negatively regulates S100A2 expression in vivo [49]. These findings suggest that elevated S100A2 expression seems to be associated with an unfavorable prognosis in brain-derived tumors.

## 9. Thyroid Cancer

Papillary carcinoma is the most common type of well-differentiated thyroid malignancy, and the representative oncogenic basis of this tumor is radiation exposure in vivo [50]. Although 89.5% of thyroid papillary carcinomas show positive expression of S100A2 in vivo [51], the detailed prognostic impact of S100A2 in thyroid papillary carcinoma remains unclear.

## 10. Lung Cancer

Pulmonary fibrosis is a progressive and sometimes fatal disease with an unfavorable prognosis that has the potential to develop into lung cancer in vivo [52]. S100A2 plays an important role in cancer progression. The downregulation of S100A2 suppresses TGF-β1-mediated epithelial–mesenchymal transition (EMT) in lung adenocarcinoma cells in vitro [53]. Furthermore, epidermal growth factor (EGF)-stimulated EGF receptor (EGFR) phosphorylation induces the expression of S100A2 in nonsmall-cell lung carcinoma (NSCLC) in vitro [54]. Consistently, the overexpression of S100A2 in NSCLC tumor cells enhances transendothelial migration and distant organ metastasis in vivo [55]. 

These findings show that S100A2 may play a dominant role as a positive driver of tumor development in NSCLC. Positive S100A2 expression is significantly related to a high frequency of lymph node metastasis in lung adenocarcinoma in vivo [56]. In addition, high levels of S100A2 mRNA expression are related to poor clinical survival in patients with NSCLC undergoing surgical resection in vivo [57]. High expression of S100A2 is associated with a significantly unfavorable overall survival and disease-specific survival rate after surgery in patients with NSCLC in vivo [58].

In contrast, S100A2 expression is associated with better prognostic outcomes in patients with p53-negative tumors in lung adenocarcinoma in vivo [59], suggesting that S100A2 and other oncogenic factors might be involved in the pathogenesis of NSCLC.

## 11. Renal Cell Carcinoma

Renal cell carcinoma is the most common kidney cancer in adults, and S100A2 expression is decreased due to the DNA methylation of the promoter site of *S100A2* in vitro [60]. Although the prognostic influence remains unclear, lower S100A2 expression in renal cell carcinoma might be expected to be advantageous for tumor development.

## 12. Liver Diseases

Hepatocellular carcinoma (HCC) is a representative common hepatic cancer, and S100A2 is highly expressed and associated with advanced clinical features in HCC patients in vivo [10,61]. High S100A2 expression is associated with an unfavorable overall survival in vivo [10,62]. As S100A2 expression is upregulated, the hypomethylation status of the CpG island located at the *S100A2* promoter site is correlated with the induction of S100A2 expression by HCC cells in vitro [10].

## 13. Breast Cancer

S100A2 expression decreases in advanced breast cancer in vivo [61]. S100A2 is expressed in 37% of specimens of carcinoma in situ, and in <15% of breast cancer samples in vivo [63], suggesting that the loss of S100A2 is related to the development of breast cancer and does not appear to be with an early phase of tumor development. Highly metastatic breast cancers exhibit elevated levels of S100A2 in vivo [64]. miRNAs are involved in the epigenetic regulation of S100A2 expression in breast cancer cells. miR-325-3p overexpression has similar effects to S100A2 silencing in breast cancer tumor cells in vitro [65]. miR-325-3p overexpression in breast cancer tumor cells enhances tumor cell proliferation and invasion, and EMT development by suppressing S100A2 expression in vitro [65].

## 14. Bladder Cancer

S100A2 is associated with the development of bladder cancer in vivo [66]. Decreased expression of S100A2 is related to tumor progression and unfavorable clinical outcomes in patients with bladder cancer in vivo [66].

## 15. Gynecologic Cancers

S100A2 expression is upregulated in patients with ovarian cancer, and high S100A2 expression is associated with advanced clinical stage and unfavorable prognosis in vivo [67,68,69].

S100A2 expression is upregulated in endometrial carcinoma tissues in vivo [70]. Patients with endometrial carcinoma with high S100A2 expression exhibit poor overall survival and disease-specific survival in vivo [70].

## 16. Pancreatic Cancer

Pancreatic cancer is an aggressive malignant tumor with a high mortality rate despite current advances in therapeutic approaches [71]. S100A2 is associated with immunological phenotypes in the tumor microenvironment of pancreatic cancer. S100A2 expression shows a relationship with lower abundance of CD8^+^ T cells, activated natural killer (NK) cell infiltration, and a higher abundance of M0 macrophage involvement in vivo [11]. S100A2 is an unfavorable prognostic indicator in pancreatic cancer, and S100A2 expression is positively correlated with the expression of programmed cell death 1-ligand 1 (PD-L1) in pancreatic cancer cells in vivo [11]. Although high S100A2 expression represents an independent predictor of survival in patients with pancreatic cancer, patients with S100A2-negative tumors present favorable survival as patients undergoing pancreatectomy, even in the presence of involved positive surgical margins or lymph node metastases in vivo [72]. Pancreatic adenocarcinoma patients with high S100A2 expression exhibit poor prognosis in vivo [73,74,75]. As a mechanism of downregulating S100A2, the promoter methylation of *S100A2* is involved in the development of pancreatic cancer in vitro [74].

## 17. Melanoma

S100A2 is highly expressed in primary melanoma, whereas its expression is low in metastatic melanoma cells in vivo [76]. S100A2 enhances the antitumor action of treatment with retinoid and thiazolidinedione against melanoma in vitro [77]. Although S100A2 is a ligand of the receptor for advanced glycation end products (RAGEs) [14], which participate in melanoma progression by promoting tumor growth, anti-RAGE antibody treatment reduces tumor growth in vitro [78], S100A2 might have other action points in melanoma to exert antitumor action against melanoma.

## 18. Prostate Cancer

Lower S100A2 expression is associated with favorable clinical outcomes in prostate cancer in vivo [79,80]. Although benign prostate hypertrophy and prostatitis display increased S100A2 expression, low-grade prostate cancer exhibits reduced expression of S100A2 in vivo [79,80]. DNA methylation has been postulated as a mechanism underlying the downregulation of S100A2 in prostate cancer, and *S100A2* methylation has also been observed in 75% of nonmalignant tissues and 100% of benign prostate hypertrophy cases. Immunostaining analysis reveals loss of S100A2 expression in prostate cancer in vivo [81].

## 19. Squamous Cell Carcinoma

S100A2 expression appears to differ from that at the original site of squamous cell carcinoma. Most squamous cell carcinomas are associated with an unfavorable prognosis, depending on the reduced expression of S100A2. S100A2 expression has been observed in various squamous cell carcinomas, including those of conjunctival origin [82].

S100A2 expression in laryngeal squamous cell carcinoma is positively correlated with longer relapse-free and overall survival [83]. These findings indicate that S100A2 acts as a tumor suppressor. A high frequency of metastasis is found in patients with S100A2-negative oral squamous cell carcinoma tumors in vivo [84].

Another study revealed that, in primary squamous cell carcinoma of the larynx, the high expression of S100A2 appeared to be a significant independent predictive factor for favorable survival in vivo [85]. Another study showed that, in esophageal squamous cell carcinomas, S100A2 expression is significantly higher in well-differentiated tumors and lower lymph node metastasis samples in vivo [86], while S100A2-overexpressing tumor cells exhibit a high frequency of lymph node and distant metastases in esophageal squamous cell carcinoma in vivo [87]. Consistently, another study showed that the expression of S100A2 protein is correlated with tumor differentiation and lymph node metastasis in vivo [88]. Studies of patients with oral squamous cell carcinoma also indicate that S100A2 is related to tumor recurrence in vivo [89]. Cisplatin is an important drug for the treatment of head and neck squamous cell carcinoma in vivo [90]. S100A2 was identified as a potential cisplatin-specific chemoresistance factor in vivo [90]. Silencing S100A2 expression in head and neck squamous cell carcinoma reveals cisplatin sensitivity in acquired and naturally cisplatin-resistant tumor cells in vivo [90].

Regarding S100A2 action in squamous cell carcinoma, S100A2 negatively regulates cell motility in squamous cell carcinoma in vivo [91]. Ectopic expression of S100A2 in the human malignant squamous cell carcinoma cell line KB results in significant inhibition of proliferation, migration, and invasion, which can be negatively regulated by cyclooxygenase-2 (COX-2) expression in vitro [92].

Although it is necessary to clarify the detailed molecular mechanisms involving S100A2 in squamous cell carcinoma, the spectrum of action of S100A2 might have antitumor effects on the development of squamous cell carcinoma.

## 20. Gastroenterological Cancers

Gastric cancer is a common type of gastrointestinal cancer, and S100A2 expression is downregulated in gastric cancer in vivo [93]. S100A2 downregulation is significantly associated with poor differentiation, tumor invasion, and lymph node metastasis in vivo [93,94,95], and unfavorable survival in patients with gastric cancer in vivo [93,94]. S100A2 acts as a tumor suppressor in gastric cancer, and it suppresses the extracellular-signal-regulated kinase (ERK) and mitogen-activated protein kinase (MAPK)/ERK kinase (MEK) signalling pathway, which is essential for the development of gastric cancer. Consistently, the activation of this signaling pathway by S100A2 downregulation increases tumor invasion in gastric cancer cells in vitro [93].

The overexpression of S100A2 in Barrett’s adenocarcinoma was observed showing a tendency for well-differentiated tumors in vivo [96].

Elevated S100A2 expression is associated with unfavorable clinical survival in colorectal cancer in vivo [97,98,99] and tumor recurrence in vivo [100]. S100A2 overexpression enhances glucose metabolism and cell proliferation owing to the advantage of tumor development in vivo [101]. S100A2 activates the phosphoinositide 3-kinase (PI3K)/AKT serine/threonine kinase (AKT) signaling pathway and upregulates GLUT1 expression, which induces glycolytic reprogramming and consequently increases the proliferation of colorectal cancer cells in vitro [101].

S100A2 expression in cholangiocarcinoma cells is related to a high frequency of lymph node metastasis, advanced clinical stage, and poor patient survival rates in vivo [102].

## 21. Summary of S100A2 Regulation

The role of S100A2 in inflammatory diseases is largely unknown. However, S100A2 is expected to be a positive driver for the development of inflammatory reactions in atopic dermatitis, psoriasis, drug eruption, and asthma.

High S100A2 expression is associated with an unfavorable clinical course in brain tumors, such as low-grade glioma and glioblastoma, NSCLC, hepatocellular carcinoma, ovarian cancer, endometrial carcinoma, pancreatic cancer, prostate cancer, colorectal cancer, and cholangiocarcinoma. In contrast, lower S100A2 expression is associated with an unfavorable clinical course in patients with renal cell carcinoma, breast cancer, bladder cancer, melanoma, and gastric cancer. These findings indicate that the prognostic impact of S100A2 is completely different in each malignancy. As one of the reasons, S100A2 was a responsible for tumor cell migration in some tumor cells in vitro [91], suggesting that A100S2 might have different unknown action mechanisms in each tumor.

The role of S100A2 in benign tumors is also unknown; however, its expression can also be used as a marker to distinguish it from other benign or malignant tumors.

## 22. Conclusions

S100A2 expression and its roles are completely different in different diseases. Our review shows that S100A2 functions as a tumor suppressor; however, the influence of its expression is completely different in different types of malignant tumors. In particular, there exists a need to elucidate expression alterations of S100A2 in early and advanced stages of malignant tumors, in addition to determining the actual impact of S100A2 on their prognosis. Despite the analysis of S100A2 in cancers, there are only a few studies focused on inflammatory diseases and benign tumors. In particular, the immunomodulatory action of S100A2 has been speculated upon in previous studies, and further investigation is necessary to clarify the actual role of immunomodulatory functions of S100A2 in inflammatory diseases. In addition, a limited number of studies reported the role of A100A2 as a biomarker in malignancies and inflammatory diseases to estimate the prognosis or disease severity [12,103].

Taken together, we summarized the current trend of the knowledge gained from recent studies that showed a clear role of S100A2 in certain malignancies; however, the unknown mechanisms of S100A2 should be elucidated by further analysis in the future.

This figure shows the regulatory mechanism and intracation factors to enhance the action of S100A2 in physiological and pathological conditions. Various factors are involved in S100A2 regulation for physiological and pathological actions. TGF-β, TNF-α, and p53 can enhance the induction of S100A. S100A2 is also regulated by epigenetic modifications. DNA methylation impairs S100A2 expression, while histone methylation accelerates S100A2 gene expression. S100A2 also involves interaction with other molecules, such as S100A4, EGFR, DeltaNp63, and erythropoietin for the enhancement of the ability of S100A2 function.

The prognostic impact or the severity of influences is summarized in this figure. Although S100A2 has a tumor suppressor function, the recently updated research focusing on the prognostic impact of S100A2 revealed the various influences of S100A2 in human malignancies in addition to inflammatory diseases. High S100A2 expression was associated with a better prognosis in patients with breast cancer, bladder cancer, melanoma, laryngeal squamous cell carcinoma, and gastric cancer. On the other hand, a high expression of S100A2 was associated with a poorer prognosis in patients with nonsmall-cell lung cancer, hepatocellular carcinoma, ovarian cancer, endometrial carcinoma, pancreatic cancer, prostate cancer, oral squamous cell carcinoma, colorectal cancer, cholangiocarcinoma. S100A2 was also involved in the development of inflammatory reactions, atopic dermatitis, drug eruption, psoriasis, delayed wound healing, and asthma.

## Data Availability

Not applicable.
